# Analysis of genetic characteristics of pig breeds using information on single nucleotide polymorphisms

**DOI:** 10.5713/ajas.18.0304

**Published:** 2018-08-27

**Authors:** Sang-Min Lee, Jae-Don Oh, Kyung-Do Park, Kyoung-Tag Do

**Affiliations:** 1 Department of Animal Biotechnology, Chonbuk National University, Jeonju 54896, Korea; 2 Department of Animal Biotechnology, Jeju National University, Jeju 63243, Korea

**Keywords:** Effective Population Size, F-statistics, Heterozygosity, Linkage Disequilibrium, Polymorphism Information Content

## Abstract

**Objective:**

This study was undertaken to investigate the genetic characteristics of Berkshire (BS), Landrace (LR), and Yorkshire (YS) pig breeds raised in the Great Grandparents pig farms using the single nucleotide polymorphisms (SNP) information.

**Methods:**

A total of 25,921 common SNP genotype markers in three pig breeds were used to estimate the expected heterozygosity (*H_E_*), polymorphism information content, F-statistics (*F_ST_*), linkage disequilibrium (LD) and effective population size (N_e_).

**Results:**

The chromosome-wise distribution of *F_ST_* in BS, LR, and YS populations were within the range of 0–0.36, and the average *F_ST_* value was estimated to be 0.07±0.06. This result indicated some level of genetic segregation. An average LD (r^2^) for the BS, LR, and YS breeds was estimated to be approximately 0.41. This study also found an average N_e_ of 19.9 (BS), 31.4 (LR), and 34.1 (YS) over the last 5th generations. The effective population size for the BS, LR, and YS breeds decreased at a consistent rate from 50th to 10th generations ago. With a relatively faster N_e_ decline rate in the past 10th generations, there exists possible evidence for intensive selection practices in pigs in the recent past.

**Conclusion:**

To develop customized chips for the genomic selection of various breeds, it is important to select and utilize SNP based on the genetic characteristics of each breed. Since the improvement efficiency of breed pigs increases sharply by the population size, it is important to increase test units for the improvement and it is desirable to establish the pig improvement network system to expand the unit of breed pig improvement through the genetic connection among breed pig farms.

## INTRODUCTION

An investigation of the genetic architecture is the first important step towards genomic selection for the improvement of pig breeds. Today, the genetic information on breeding pigs has been accumulating. If the reference population is entirely established in the future, genomic selection can be possible and used to increase selection accuracy through the use of genomic and phenotypic data along with pedigree information [1].

A comprehensive information on the genetic diversity and introgression is essential for an improvement of national breeding as well as the design of conservation programs. In the past, the genetic diversity in pigs was mostly reported using information on both microsatellite markers [2,3] and mtDNA [4]. However, the advantages of single nucleotide polymorphism (SNP) over microsatellite or mtDNA are that they represent the major source of genetic variation, show low mutation rates, and are associated with complex heritable traits [5]. Nowadays, thousands of SNP information are readily available, with the advent of next generation sequencing technology [6]. Through various high-density SNP panels, the Illumina Porcine 60k Bead Chip allows for more precise and comprehensive genome-wide investigation of genetic diversity, and the degree of admixture among pig breeds [7–9].

Linkage disequilibrium (LD), on the other hand, existing within population could assist in determining the relationship among the SNPs which affect the economic traits, mapping the quantitative trait locus (QTL), and selecting the tagging SNP. Additionally, the LD between SNP among specific physical distance can be used to estimate the effective population size, and to identify the genetic diversity through genetic characteristics [10]. Furthermore, the QTLs governing pig economic traits have been studied frequently, primarily through genome-wide association studies using single marker regression [11–13].

This experiment was conducted to investigate the genetic characteristics and effective population sizes of Berkshire, Landrace, and Yorkshire pig breeds raised in the great grandparent (GGP) farms using the SNP information.

## MATERIALS AND METHODS

### Description of single nucleotide polymorphism data

A total of 3,710 pigs of consisting of the Berkshire (1,615), Landrace (1,041), and Yorkshire (1,054) were genotyped using Porcine SNP 60k and 61,565 SNP were collected.

To ensure the quality of the genotypic data, SNP on the sex chromosomes, SNP without information on chromosome, SNP with higher than 10% of missing rate, SNP without polymorphism (all homo or hetero), SNP with less than 1% of minor allele frequency and SNP with more than 23.93 (p<10^−6^) of Hardy-Weinberg disequilibrium chi-squared value, and animals with more than 10% of SNP missing rate were excluded from the analysis.

We found that 30, 3, and 19 pigs in Berkshire, Landrace and Yorkshire breeds had an SNP missing rate higher than 10%, respectively. Therefore, the number of pigs (SNP) after quality control in Berkshire, Landrace and Yorkshire were 1,585 (38,962), 1,038 (26,392), and 1,035 (40,783) pigs, respectively. In this study, only the 25,921 common SNPs among three breeds were used for analyses.

### Statistical models

#### Expected heterozygosity

The expected heterozygosity (*H_E_*) of a locus is defined as the probability that an individual is heterozygous in the population.

$$H_{E}=1-\sum_{i=1}^{N}p_{i}^{2}$$

where *p_i_* is the frequency of the *i*th allele of the n alleles [14].

#### Polymorphism information content

The polymorphism information content (PIC) refers to the value of a marker for detecting polymorphism with a population, depending on the number of detectable alleles and the distribution of their frequency [15].

$$PIC=1-\left(\sum_{i=1}^{n}p_{i}^{2}\right)-\sum_{i=1}^{n-1}\sum_{j=i+1}^{n}2p_{i}^{2}p_{j}^{2}$$

where *n* is the total number of alleles, *p_i_* and *p_j_* are frequency of the *i*th and *j*th alleles in the population, respectively [16]. The PIC is defined as the probability that the marker genotype of a given offspring will allow deduction, in the absence of crossing over, of which of the two marker alleles of the affected parents it received [17].

#### F-statistics (F_IS_,F_ST_,F_IT_)

The F-statistics were used for comparing the genetic characteristics among the breeds.

$$f=\frac{\left(F-\emptyset \right)}{\left(1-\emptyset \right)}$$

where *F* is the correlation of genes within individuals, *Ø* is the correlation of genes of different individuals in the same population, and *f* is the correlation of genes within individuals within populations. These parameters are related to Wright’s F-statistics [18] as: *F* = *F_IT_*, *Ø* = *F_ST_*, *f* = *F_IS_*.

For F-statistics, *F_IT_* represents the degree of genetic fixation of a breed, *F_IS_* represents the degree of inbreeding of individuals in a population, and *F_ST_* indicates the degree of genetic segregation of the populations. By using F-statistics, paired t-tests analyses with entirely SNP among breeds were performed.

*Linkage disequilibrium*:

The LD between SNP pairs was used to calculate the standardized LD value D′ [19] and r^2^ [20]. But, D′ is dependent on the frequencies of the individual alleles. Another measure of LD is r^2^, which is less dependent on allele frequencies.

The amount of LD is the value for the linkage between two different alleles and can be estimated by the standardized D (D′) [19] or r^2^ [20]. However, since the estimation of the LD using D′ can be overestimated when the population size or frequency of allele is small, it was estimated using r^2^. The measure of LD was expressed as the square (*r*^2^) of the correlation coefficient between SNP pairs, and was calculated between each allele at locus A and each allele at locus B [21]. The correlation coefficient (*r*^2^) was calculated by the formula:

$$r^{2}=\frac{D^{2}}{P_{A}P_{a}P_{B}P_{b}}$$

where D = *P_AB_*–*P_A_P_B_* and *P_A_*, *P_a_*, *P_B_*, and *P_b_* are the frequencies of alleles A, a, B, and b, respectively. *P_AB_* is the frequency of the genotype AB.

Haplotypes within haploblocks were obtained using the expectation maximization (EM) algorithm, similar to the partition/ligation [22]. When the two loci are homozygotes or one of genotypes of the two loci is homozygote, the frequency of haplotype can be calculated, but when the two loci are double heterozygotes it is difficult to distinguish the coupling (A_1_B_1_/A_2_B_2_) and repulsion (A_1_B_2_/A_2_B_1_) by the DNA chip analysis. Therefore, using the EM algorithm [22] that determines maximum likelihood estimates for the parameters in the probability model which depends on the invisible potential variables, conditional probabilities for coupling (A_1_B_1_/A_2_B_2_) and repulsion (A_1_B_2_/A_2_B_1_) were calculated and the value of LD was estimated through the repeated arithmetic calculation until the amount of change reaches less than 10^−5^ [23].

*Effective population size*:

The effective population size was determined based on a simple expectation from the amount of LD and a given chromosome segment. Since LD breaks down more rapidly over the generations for loci that are further apart, LD at large distances reflects *N_e_* at recent generations [24].

$$N_{e}=\frac{{\left(r_{c}^{2}\right)}^{-1}-1}{4c}$$

where, *N_e_* is the effective population size *t* generations ago, *c* is the recombination distance between the SNP in Morgan, c = (1/2*t*), $r_{c}^{2}$ is the mean value of *r*^2^ for markers that are *c* Morgan apart. It was assumed that 1 cM of physical distance and 1 Mb of genetic distance were identical.

## RESULTS AND DISCUSSION

### Expected heterozygosity

**Figure 1:**
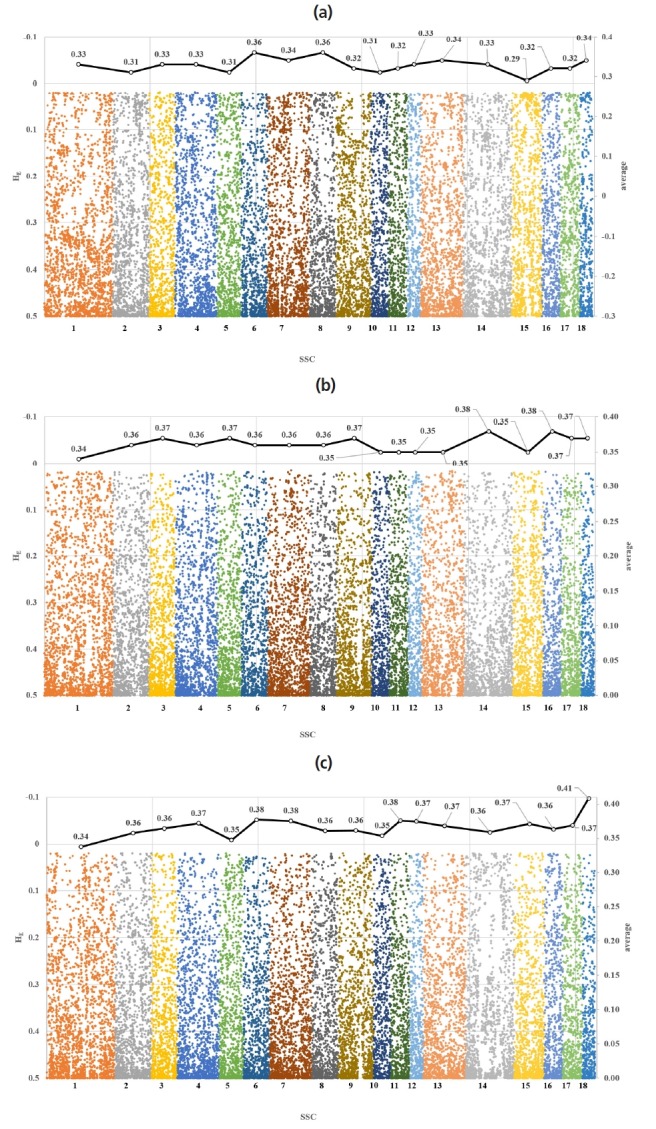
Chromosome-wise distribution of single nucleotide polymorphism heterozygosity (dots) and their average (solid line) in Berkshire (a), Landrace (b) and Yorkshire (c).

Figure 1 illustrates the distribution of expected heterozygosity for chromosome-wise SNP in the studied pig breeds. All three pig breeds showed a similar trend in the *H_E_* estimates. The estimates of the average *H_E_* in the Berkshire, Landrace, and Yorkshire were 0.33±0.15, 0.36±0.14, and 0.36±0.14, respectively. While the estimates of the average *H_E_* were low in Berkshire, they were the same in Landrace and Yorkshire. Ai et al [25] reported that research regarding genetic diversity of 18 pig breeds using 60K SNP Chip showed the similar expected heterozygosity (0.38) of Landrace and Large White. The results of this study indicated the same about expected heterozygosity. The estimates of the average *H_E_* were found to be highest in Sus scrofa chromosome 6 (SSC6 (0.36±0.15) of Berkshire, SSC18 (0.38±0.12) of Landrace, and SSC14 (0.38± 0.13) and SSC16 (0.38±0.13) of Yorkshire. On the other hand, the lowest *H_E_* estimates, in contrast, were found in SSC15 (0.29± 0.16), SSC10 (0.34±0.13), and SSC1 (0.34±0.14) in Berkshire, Landrace, and Yorkshire pigs (Figure 1).

### Polymorphism information contents

The estimates of PIC obtained using the *H_E_* values represented polymorphism information on each gene locus [16]. The estimates of the average PIC in Berkshire, Landrace, and Yorkshire breeds were 0.26±0.11, 0.28±0.10, and 0.29±0.10, respectively.

Across the chromosomes, the estimates of PIC for the SNP was highest in SSC6 (0.28±0.10) of Berkshire, SSC18 (0.30± 0.08) of Landrace, and SSC14 and 16 (0.30±0.09) of Yorkshire. On the other hand, the lowest values of PIC were observed for SSC15 (0.23±0.12) in Berkshire, for SSC10 (0.27±0.09) in Landrace, and for SSC1 (0.27±0.10) in Yorkshire. Overall, the estimates of PIC were lower than those of the average *H_E_* (Table 1).

### Pairwise t-test

Using the estimates of the average *H_E_* and PIC in each breed, pairwise t-tests were performed, across the breeds.

For the *H_E_* estimates, there was no significant (p<0.05) difference in SSC1 and SSC8 in the comparison between Berkshire and Landrace, and in SSC6 and SSC8 in the comparison between Berkshire and Yorkshire, and in SSC2, SSC3, SSC8, SSC10, SSC12, SSC15, and SSC17 in the comparison between Landrace and Yorkshire. For the PIC estimates of the average, there was no significant (p<0.05) difference in SSC1 and SSC8 in the comparison between Berkshire and Landrace, and in SSC6 and SSC8 in the comparison between Berkshire and Yorkshire, and in SSC1, SSC2, SSC3, SSC8, SSC10, SSC12, and SSC17 in the comparison between Landrace and Yorkshire (Table 2).

However, the pairwise t-tests using all SNP revealed significant differences (p<0.01) in the estimates of the average *H_E_* and PIC among Berkshire, Landrace, and Yorkshire breeds (Table 2). According to the study of Edea et al [26], the *H_E_* estimate was reported to be lowest in Berkshire breed (0.31±0.17), highest in Landrace breed (0.42±0.22), while that of Yorkshire breed was reported to be 0.35±0.17. The results of this study were consistent with those of the study [26], and the estimates of expected heterozygosity were observed the same pattern (Berkshire, 0.327±0.017; Landrace, 0.363±0.012; and Yorkshire: 0.361±0.011).

### F-statistics

**Figure 2:**
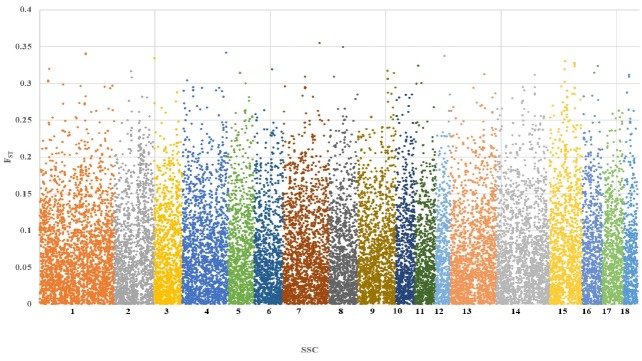
Chromosome-wise FST estimates of single nucleotide polymorphisms in Berkshire, Landrace and Yorkshire.

To investigate differences in the genetic characteristics, F-statistics were estimated among Berkshire, Landrace, and Yorkshire populations. The estimates of *F_ST_* by chromosome among breeds were in the range of 0 to 0.36, and the distributions of *F_ST_* for chromosome-wise SNP were shown Figure 2. Previous study showed that *F_ST_* among Berkshire, Landrace, and Yorkshire breeds are 0.22 for Berkshire vs Landrace, 0.24 for Berkshire vs Yorkshire, and 0.20 for Landrace vs Yorkshire [26]. As the *F_ST_* value by chromosome among breeds increased, the frequency of SNP definitely decreased, and the same trend was shown in all chromosomes.

When the *F_ST_* value among breeds was less than 0.05, the number of SNPs was 12,008 (46.3%), while it was 12,901 (49.8%) when the *F_ST_* value was between 0.05 and 0.2. Also, when the *F_ST_* value among breeds was more than 0.2, it was 1,012 (3.9%). The average *F_ST_* in all chromosomes was 0.07±0.06. This result indicated that some genetic segregation has occurred partly.

### Linkage disequilibrium

The average physical distance between adjacent SNP pairs by chromosome was largest in SSC6 (126.59 kb), smallest in SSC14 (66.73 kb) and the overall average distance was 94.09 kb (Table 3). A total of 22,571,445 SNP pairs were used to estimate LD (r^2^). The estimates of the average r^2^ between adjacent SNP were 0.411, 0.408, and 0.413 in Berkshire, Landrace, and Yorkshire, respectively. Similar results were reported in Landrace, Yorkshire, Hampshire and Duroc in the USA and their estimates were 0.36, 0.39, 0.44, and 0.46 [27]. However, Uimarie and Tapio [28] reported that their estimates were 0.47 (Yorkshire) and 0.49 (Landrace) in Finland, which were higher than those of our results. Across the chromosomes, the estimate for the r^2^ between adjacent markers was highest in SSC1 of the Berkshire breed (0.47), SSC14 of the Landrace breed (0.49), and SSC1, SSC13 and SSC14 (0.47) of the Yorkshire breed (Table 4).

**Figure 3:**
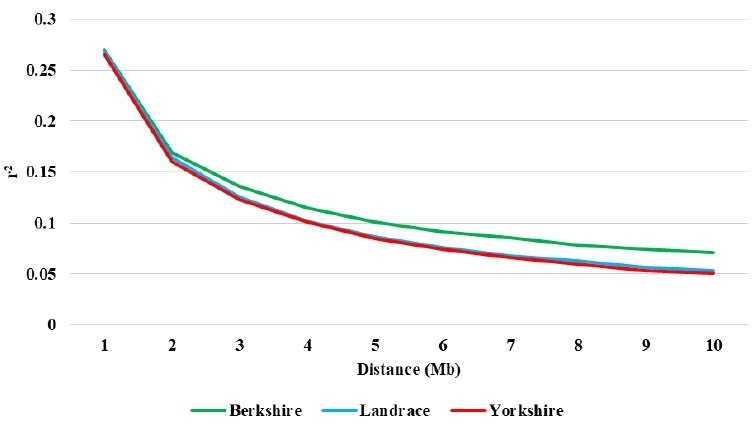
Changes in linkage disequilibrium estimates (r^2^) between single nucleotide polymorphism markers within 10 mega base (Mb) pair distance.

The values of r ^2^ decreased with increasing distance between SNP pairs (Figure 3) and the most rapid decline was observed over the first 2 Mb. But r^2^ decreased more slowly with increasing distance and was constant after 5 Mb of distance [28]. In each breed, the pattern and magnitude LD decline with distance at less than 10 Mb were almost similar.

### Effective population size

**Figure 4:**
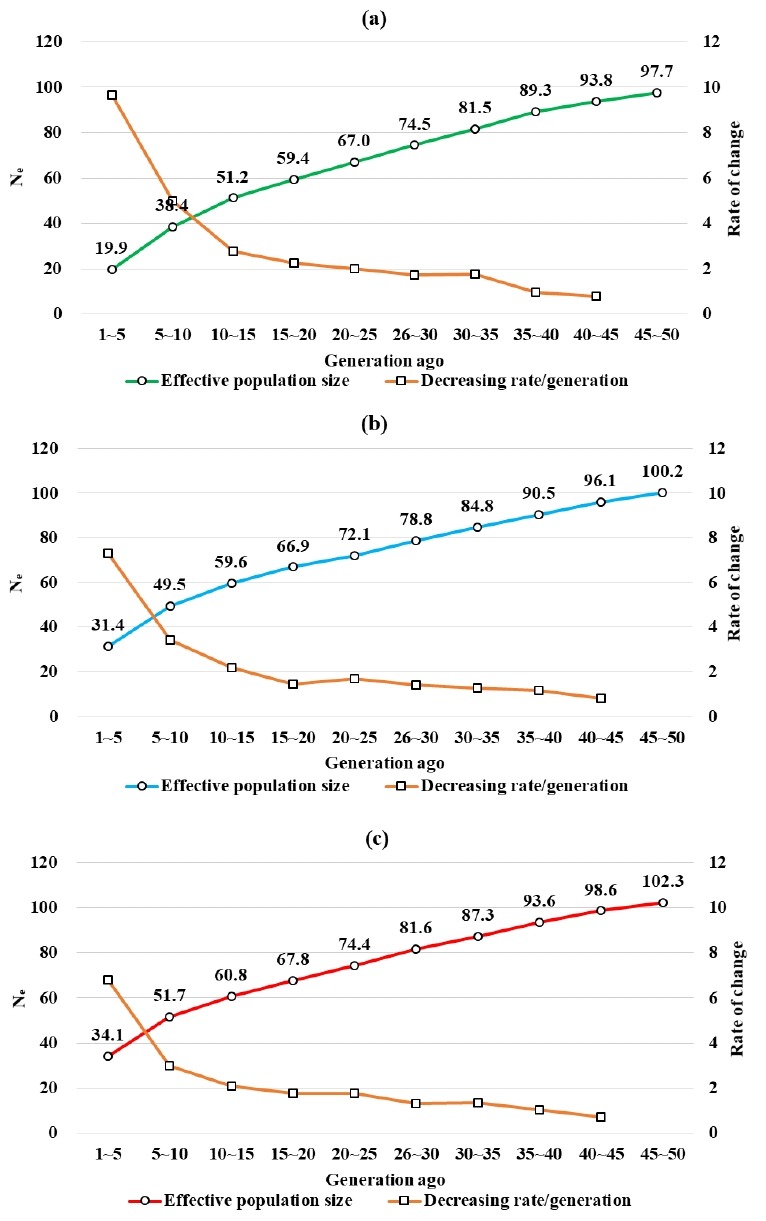
Changes in past effective population size (N_e_) in Berkshire (a), Landrace (b) and Yorkshire (c).

It can be predicted that when the LD (*r*^2^) between SNP located within close physical distances is low, genetic recombination at that locus occurred a long time ago. Similarly, when the r^2^ between SNP located within far physical distances is high, genetic recombination at that locus occurred recently. The extent of genetic recombination can be estimated by the population size, while the *N_e_* across the generations can be estimated from the *r*^2^ [10,29]. The *N_e_* for the Berkshire, Landrace, and Yorkshire over 1st–5th generation was estimated to consist of 19.87, 31.41, and 34.09 pigs, respectively (Figure 4). It was reported in a previous study that the *N_e_* of the Landrace and Yorkshire in Finland consists of approximately 80 and 55 pigs, respectively [28].

The effective population size was estimated small compared to those of the advanced countries in pig industry since the scales of domestic GGP farms were relatively small. Additionally, closed herds have been maintained and inbreeding mating system have been applied.

In Berkshire, the size of past *N_e_* from 50th to 5th generations ago had changed noticeably, from 97.7 to 50, with a gradual increase in declining rate per generation (0.8% to 9.7%). Similarly, *N_e_* declines were also observed in Landrace (100.2 to 50) and Yorkshire (102.3 to 34.1) pigs, followed by a somewhat similar declining rate. The *N_e_* for the Berkshire, Landrace, and Yorkshire decreased at constant slope from 50th generations ago to 10th generations ago, with a sharp decrease in the recent 10th generations. Similar results were reported in a study by Uimari and Tapio [28]. From these results, the intensive artificial selection seemed to be made from recent 10th generations (Figure 4).

## CONCLUSION

In order to develop customized chips for the genomic selection of various breeds, it is important to select and utilize SNP based on the genetic characteristics of each breed. Since the improvement efficiency of breed pigs increases sharply by the population size, it is important to increase test units for the improvement and it is desirable to establish the pig improvement network system to expand the unit of breed pig improvement through the genetic connection among breed pig farms.

## Figures and Tables

**Table 1:** Chromosome-wise polymorphism information content (PIC) in Berkshire (BS), Landrace (LR), and Yorkshire (YS) pigs

SSC	BS	LR	YS
1	0.27±0.14	0.27±0.10	0.27±0.10
2	0.25±0.11	0.28±0.10	0.28±0.09
3	0.26±0.11	0.29±0.09	0.29±0.09
4	0.26±0.11	0.29±0.09	0.28±0.10
5	0.25±0.11	0.27±0.11	0.29±0.09
6	0.28±0.10	0.29±0.09	0.29±0.09
7	0.27±0.11	0.29±0.09	0.29±0.10
8	0.28±0.11	0.28±0.10	0.29±0.09
9	0.26±0.11	0.28±0.10	0.29±0.09
10	0.25±0.11	0.27±0.09	0.28±0.10
11	0.26±0.11	0.29±0.09	0.28±0.09
12	0.26±0.11	0.28±0.10	0.28±0.10
13	0.27±0.10	0.29±0.09	0.28±0.10
14	0.26±0.11	0.28±0.10	0.30±0.09
15	0.23±0.12	0.29±0.09	0.28±0.10
16	0.26±0.10	0.28±0.10	0.30±0.09
17	0.26±0.10	0.28±0.10	0.29±0.10
18	0.27±0.11	0.30±0.08	0.29±0.09
Overall	0.26±0.11	0.28±0.10	0.29±0.10

SSC, Sus scrofa chromosome.

**Table 2:** Chromosome-wise mean differences1) for heterozygosity (*H_E_*) and polymorphism information content (PIC) among Berkshire (BS), Landrace (LR), and Yorkshire (YS) breeds

SSC	*H_E_* ((|D| )			PIC ((|D| )		
	
	BS vs LR	BS vs YS	LR vs YS	BS vs LR	BS vs YS	LR vs YS
1	0.0049^NS^	0.0119**	0.0069**	0.0041^NS^	0.0078**	0.0036^NS^
2	0.0487**	0.0487**	0.001^NS^	0.0349**	0.0362**	0.0013^NS^
3	0.0366**	0.0438**	0.0073^NS^	0.0265**	0.0319**	0.0054^NS^
4	0.0383**	0.0258**	0.0125**	0.0285**	0.0196**	0.0089**
5	0.0304**	0.0523**	0.0219**	0.0209**	0.0376**	0.0167**
6	0.0141**	0.0029^NS^	0.0113*	0.0106**	0.0031^NS^	0.0075*
7	0.0321**	0.0243**	0.0078*	0.0238**	0.0181**	0.0057*
8	0.0013^NS^	0.0071^NS^	0.0083^NS^	0.0001^NS^	0.0068^NS^	0.0067^NS^
9	0.381**	0.0515**	0.0134**	0.0274**	0.0366**	0.0092**
10	0.0273**	0.0380**	0.0107^NS^	0.0218**	0.0281**	0.0063^NS^
11	0.0450**	0.0332**	0.0118*	0.0335**	0.0251**	0.0084*
12	0.0282**	0.0187*	0.0094^NS^	0.0208**	0.0144**	0.0065^NS^
13	0.0190**	0.0102*	0.0088*	0.0133**	0.0069**	0.0064*
14	0.0245**	0.0478**	0.0233**	0.0169**	0.0335**	0.0166**
15	0.0754**	0.0656**	0.0098^NS^	0.0553**	0.0484**	0.0069
16	0.0219**	0..0600**	0.0382**	0.0143**	0.0414**	0.0271**
17	0.0288**	0.0515**	0.0227**	0.0198**	0.0342**	0.0144**
18	0.0398**	0.0306**	0.0092^NS^	0.0298**	0.0229**	0.0068^NS^
Overall	0.0289**	0.0327**	0.0038**	0.0211**	0.0236**	0.0144**

SSC, Sus scrofa chromosome; NS, not significant.

1) Differences were inferred based on pairwise T test.

* p<0.05,

** p<0.01.

**Table 3:** Chromosome-wise number of SNP, SNP pairs and average distance between adjacent marker pairs (ADAM, kb) among three pig breeds

SSC	No. of SNP	No. of SNP pairs	ADAM (kb)
1	3,233	5,224,528	97.26
2	1,728	1,492,128	93.73
3	1,218	741,153	117.26
4	1,996	1,991,010	71.86
5	1,126	633,375	98.44
6	1,243	771,903	126.59
7	1,994	1,987,021	67.41
8	1,252	783,126	117.72
9	1,656	1,370,340	92.56
10	827	341,551	93.91
11	885	391,170	98.55
12	651	211,575	97.39
13	1,999	1,997,001	108.92
14	2,302	2,648,451	66.73
15	1,414	998,991	111.24
16	872	379,756	99.54
17	929	431,056	74.66
18	596	177,310	100.73
Overall	25,921	22,571,445	94.09

SNP, single nucleotide polymorphism; ADAM, average distance between adjacent marker; SSC, Sus scrofa chromosome.

**Table 4:** Mean linkage disequilibrium (r2) estimates for adjacent (ADJ) and all pairs (ALL) of SNP in Berkshire (BS), Landrace (LR), and Yorkshire (YS)

SSC	BS		LR		YS	
		
	All	ADJ	All	ADJ	All	ADJ
1	0.05	0.47	0.02	0.46	0.02	0.47
2	0.04	0.40	0.03	0.39	0.03	0.39
3	0.04	0.38	0.03	0.39	0.03	0.34
4	0.03	0.42	0.02	0.41	0.02	0.43
5	0.03	0.35	0.02	0.35	0.02	0.35
6	0.03	0.38	0.03	0.40	0.03	0.43
7	0.03	0.40	0.03	0.39	0.02	0.38
8	0.04	0.39	0.02	0.37	0.03	0.40
9	0.04	0.36	0.03	0.38	0.03	0.42
10	0.03	0.34	0.02	0.37	0.02	0.32
11	0.04	0.39	0.03	0.35	0.02	0.33
12	0.04	0.41	0.03	0.37	0.03	0.36
13	0.04	0.41	0.04	0.41	0.04	0.47
14	0.05	0.46	0.04	0.49	0.03	0.47
15	0.03	0.44	0.02	0.43	0.02	0.43
16	0.04	0.39	0.03	0.39	0.04	0.39
17	0.04	0.41	0.03	0.37	0.03	0.40
18	0.05	0.43	0.04	0.38	0.03	0.36
Overall	0.04	0.41	0.03	0.41	0.03	0.41

SSC, Sus scrofa chromosome.
